# Stereotypical Chronic Lymphocytic Leukemia B-Cell Receptors Recognize Survival Promoting Antigens on Stromal Cells

**DOI:** 10.1371/journal.pone.0015992

**Published:** 2010-12-30

**Authors:** Mascha Binder, Barbara Léchenne, Ramesh Ummanni, Christan Scharf, Stefan Balabanov, Maria Trusch, Hartmut Schlüter, Ingke Braren, Edzard Spillner, Martin Trepel

**Affiliations:** 1 Department of Oncology and Hematology, Hubertus Wald Tumorzentrum/University Cancer Center Hamburg, University Medical Center Hamburg-Eppendorf, Hamburg, Germany; 2 Department of Otorhinolaryngology, University of Greifswald, Greifswald, Germany; 3 Department of Clinical Chemistry, University Medical Center Hamburg-Eppendorf, Hamburg, Germany; 4 Department of Chemistry, University of Hamburg, Hamburg, Germany; University of Bergen, Norway

## Abstract

Chronic lymphocytic leukemia (CLL) is the most common leukemia in the Western world. Survival of CLL cells depends on their close contact with stromal cells in lymphatic tissues, bone marrow and blood. This microenvironmental regulation of CLL cell survival involves the stromal secretion of chemo- and cytokines as well as the expression of adhesion molecules. Since CLL survival may also be driven by antigenic stimulation through the B-cell antigen receptor (BCR), we explored the hypothesis that these processes may be linked to each other. We tested if stromal cells could serve as an antigen reservoir for CLL cells, thus promoting CLL cell survival by stimulation through the BCR. As a proof of principle, we found that two CLL BCRs with a common stereotyped heavy chain complementarity-determining region 3 (previously characterized as “subset 1”) recognize antigens highly expressed in stromal cells – vimentin and calreticulin. Both antigens are well-documented targets of autoantibodies in autoimmune disorders. We demonstrated that vimentin is displayed on the surface of viable stromal cells and that it is present and bound by the stereotyped CLL BCR in CLL-stroma co-culture supernatant. Blocking the vimentin antigen by recombinant soluble CLL BCR under CLL-stromal cell co-culture conditions reduces stroma-mediated anti-apoptotic effects by 20–45%. We therefore conclude that CLL BCR stimulation by stroma-derived antigens can contribute to the protective effect that the stroma exerts on CLL cells. This finding sheds a new light on the understanding of the pathobiology of this so far mostly incurable disease.

## Introduction

Chronic lymphocytic leukemia (CLL) is the most prevalent type of leukemia [1], [2]. This incurable disease most commonly consists of malignant B-cells expressing common B-cell markers as well as monoclonal membrane immunoglobulin, the B-cell receptor for antigen (BCR) [3]. Major progress has been made in understanding the functional role of the BCR as well as the microenvironment in CLL pathobiology, providing crucial insights into the biology of this cancer in recent years.

In lymphatic tissues and the bone marrow, CLL cells are in close contact with a connective tissue network of mesenchyma-derived stromal cells [4], [5], [6] including mesenchymal marrow stromal cells [7], [8], CD68+ monocyte-derived nurse-like cells (NLC)[4], and follicular dendritic cells [9]. This supportive hematopoietic microenvironment protects CLL cells from spontaneous and drug-induced apoptosis [4] and is therefore studied as a novel drug target in CLL [6], [10], [11]. The CLL-stroma contact is mediated primarily by cytokine receptors and adhesion molecules. One major cytokine axis involves the microenvironmental expression and secretion of stromal cell-derived factor-1 (SDF-1) and CXCL13 which bind to the respective cytokine receptors on CLL cells, promoting migration and survival in CLL cells. In addition to classical cytokines, stromal cells secrete hedgehog ligands, which promote survival in CLL cells, as well as a range of anti-apoptotic membrane proteins such as B-cell-activating factor of the tumor necrosis factor family (BAFF), the proliferation-inducing ligand APRIL [12], and CD31 [13]. CLL-stroma adhesion is largely mediated by integrins, particularly VLA-4 (CD49d), which attaches to stromally expressed VCAM-1 and fibronectin [14], [15], [16]. The complex cross-talk between CLL cells and their protective environment has recently been reviewed comprehensively [6].

Microenvironmental stimuli by adhesion molecules and cytokines seem not to be the only factors promoting survival of B-CLL cells. There is emerging evidence that the development and course of this disease may also be driven by antigenic stimulation through the BCR [17], [18], [19], [20], [21]. Our current understanding of the configuration of BCRs in CLL strongly supports this hypothesis. During normal B-cell development, genetic recombination of various immunoglobulin-encoding genes and somatic hypermutation shape BCRs and their highly variable complementarity-determining regions 3 (CDR3) such that each B-cell recognizes a particular antigen. If the development of the malignant CLL clone occurred independently of antigenic interaction, one would expect the gene usage and CDR3 sequences (the most individual antigen-binding part of the immunoglobulin) of CLL BCRs to be randomly distributed as in normal B-cells. However, the CLL immunoglobulin gene usage is biased [22], [23], [24], [25] and a number of highly similar CDR3 regions are expressed. Indeed, more than 26% of CLL cells express BCRs belonging to one of almost 150 stereotyped subsets with virtually identical CDR3 sequences characterized so far [19], [20], [24], [26], [27], [28]. Thus, one could postulate that at least CLL cases with stereotyped BCRs recognize a limited number of epitopes as part of certain antigens that may therefore trigger and/or sustain the disease through B-cell-receptor-mediated cell activation. Indeed, CLL BCRs react with recurring self-antigens *in vitro*, including IgG, thyroglobulin, DNA, actin, cardiolipin and others as well as with microbial antigens and epitopes exposed on cell surfaces as a result of apoptosis [29], [30], [31], [32], [33].

Although microenvironmental stimulation and antigenic drive through the BCR have been studied mainly as independent phenomena, there is some recent evidence that these processes may actually be linked in a broader concept of CLL pathogenesis and progression. Burger et al. [34] found that CLL cells upregulate the expression of the chemoattractants CCL3 and CCL4 when co-cultured with NLCs. This upregulation could be inhibited by blocking BCR signaling with a specific Syk inhibitor, suggesting that antigenic stimulation through the BCR had caused CCL3/4 upregulation under co-culture conditions. Moreover, the pro-survival effects of nurse-like cells could be abrogated by blocking BCR signaling with a Syk inhibitor [35].

Here we set out to test the hypothesis if stromal cells could serve as an antigen reservoir for CLL cells, thus promoting CLL cell survival by stimulation through the BCR. We found that CLL BCRs expressing a stereotyped heavy chain complementarity-determining region 3 (HCDR3) can recognize the antigens vimentin and calreticulin which are highly expressed in stromal cells. We showed that the cytoskeletal protein vimentin is displayed on the surface of viable NLCs and that this BCR interaction contributes to stroma-mediated anti-apoptotic effects. Our results indicate that, in addition to the known effects of survival factors and adhesion molecules, stroma-mediated protection from apoptosis can be achieved through CLL BCR stimulation by stroma-derived antigens.

## Materials and Methods

### Ethics statement

Blood samples with clinical and laboratory features of CLL were taken after written informed consent as approved by the University of Freiburg's institutional review board. Clinical investigations were conducted according to the principles expressed in the Declaration of Helsinki.

### Primary CLL cells, cell lines, culture conditions and generation of nurse-like cells

Peripheral mononuclear cells (PBMC) from the blood of patients with CLL were purified by Ficoll separation and directly used for experiments. Alternatively, PBMCs were resuspended in 90% fetal calf serum (FCS) and 10% dimethyl sulfoxide (DMSO) and stored in liquid nitrogen until use. Primary CLL cells were co-cultured on a feeder layer of M210B4 murine stromal cells in RPMI medium supplemented with 10% FCS and 1% penicillin/streptomycin for up to one week. Alternatively, transfected HEK 293T cells were used as feeder layer as described below. HEK293T cells, HeLa cells and MCF-7 cells were purchased from the American Type Culture Collection (ATCC) and cultured in DMEM medium supplemented with 10% FCS and 1% penicillin/streptomycin.

To generate nurse-like cells (NLC) from the blood of patients with CLL, purified PBMCs were resuspended in RPMI medium supplemented with 10% FCS and 1% penicillin/streptomycin at a density of 1×10^7^ cells/ml and cultured in cell culture flasks (175 cm^2^) for 14 days. Adherent NLCs appeared on the bottom of the flask between seven and 14 days of culture and could be separated from CLL cells in suspension by washes with PBS.

### Recombinant expression of BCRs as IgG1 antibody

Immunoglobulin heavy and light chain sequences of a cohort of CLL patients were determined by anchored PCR cloning into the TOPO TA vector (Invitrogen) as previously described [36], [37]. Patients with the code 014 and 044 belonged to stereotyped subset 1, 022 belonged to subset 3 and 015 belonged to subset 7 described by Stamatopoulos [19] and Murray [38] and were therefore chosen for further analysis. Variable heavy and light chain genes from patient 014, 044 and 022 were amplified from cDNA by PCR with clone-specific primers and cloned into the mammalian expression vector *pBUD opti human kappa* containing the human IgG1 kappa constant regions [39]. As the CLL clone of patient 015 expressed a lambda light chain, its variable regions were cloned into *pBUD opti human lambda* containing the human IgG1 lambda constant regions. To generate a control BCR, random variable heavy and light chain regions were cloned into *pBUD opti human kappa*. The resulting immunoglobulin was designated as IgGr. After transfection into HEK293T cells and positive selection with zeocin (100 µg/ml), recombinant BCRs (termed Ig014, Ig044, Ig022, Ig015 and IgGr) were affinity purified from supernatant using protein-A sepharose as previously described [40].

### Preparation of protein extracts from cells

Adherent cells were chemically detached from the culture flask with trypsin, washed once with PBS, pelleted and resuspended in up to 1 ml of RIPA protein extraction buffer (50 mM Tris-HCl, pH 7.4, NP-40 1%, Na-deoxycholate 0.25%, 150 mM NaCl, protease inhibitor), depending on the number of pelleted cells. After 30 minutes of incubation on ice, the protein extract was cleared from insoluble material by centrifugation at full speed in a table centrifuge. Protein concentrations were determined by Bradford assay (Bio-Rad) following the manufacturer's instructions.

### One-dimensional and two-dimensional gel electrophoresis

For one-dimensional gel electrophoresis protein extracts (5 µg), prepared as described above, were resuspended in a conventional protein sample buffer containing beta 2-mercaptoethanol as reducing agent, heated at 95°C for 5 minutes to denature proteins and loaded on a 10% SDS PAGE gel. Electrophoresis was performed using the PROTEAN® Mini Cell system (Bio-Rad) with constant voltage (80 V) at 20°C.

For two-dimensional gel electrophoresis, isoelectric focussing (IEF) was performed with 7 cm pH 4–7 IPG strips (GE Healthcare) according to the manufacturer's instructions with minor modifications to improve resolution. Briefly, 30 µg protein extracts prepared as described above were diluted to 125 µl with rehydration buffer (8 M urea, 2 M thiourea, 2% CHAPS, 50 mM DTT with 0.5% v/v IPG buffer pH 4–7, [GE Healthcare]) and used to passively rehydrate each IPG strip overnight. Proteins were separated by the PROTEAN IEF system (Bio-Rad) at 20°C. The focusing was started at 250 V for 30 minutes, and then increased to 5000 V with 5000 Vh linear gradient. The samples were maintained at 5000 V until a total run of 12 kVh. After IEF, the IPG strips were equilibrated (10 minutes) in equilibration buffer (0.375 M Tris-HCl, pH 8.8, 6 M urea, 20% glycerol, 2% SDS and 130 mM DTT) and then transferred to equilibration buffer containing 135 mM iodoacetamide (10 minutes) with constant shaking. The equilibrated strip was applied onto the top of a 12.5% SDS-PAGE gel and sealed with 1% agarose prepared in SDS-Tris-glycine buffer with trace amounts of bromophenol blue as a tracking dye to monitor electrophoresis. Electrophoresis was performed as described for one-dimensional gel electrophoresis.

Gels from one- or two-dimensional gel electrophoresis were subjected to Coomassie brilliant blue staining or to western blotting.

### Coomassie brilliant blue staining

Gels were soaked with coomasie colloidal staining solution (Serva) for 2 hours or overnight. Then gels were de-stained in a solution containing 10% methanol and 7% acetic acid to remove background staining.

### Western blotting

After electrophoresis, proteins were transferred to polyvininylidene difluoride (PVDF) membranes. After electroblotting membranes were blocked with Tris-buffered saline (TBS) 0.1% Tween 20 containing 5% nonfat dry milk and 1% BSA for 1 h at room temperature. The membranes were incubated overnight at 4°C with a recombinant CLL BCR as primary antibody (100 µg antibody/10 ml blocking buffer). After washing in TBS 0.1% Tween 20, membranes were incubated with a goat anti-human IgG antibody (Dianova) diluted 1∶20000 in blocking buffer for two hours. After a second washing step, membranes were incubated for two hours with a horseradish peroxidase-conjugated rabbit anti-goat IgG antibody. For detection of vimentin, membranes were incubated for two hours with a commercially available vimentin antibody (clone M44314F, Meridian Life science) diluted 1∶5000 in blocking buffer. followed by secondary detection with a horseradish peroxidase-conjugated anti-mouse IgG antibody (Santa Cruz Biotechnology) diluted 1∶20 000 in blocking buffer. Immunodetection was performed using the ECL western blot analysis system (GE Healthcare), followed by autoradiography on ECL Hyperfilms (GE Healthcare).

### Protein identification by MALDI TOF/TOF

Coomassie-stained two-dimensional electrophoresis gels and immunoblots were scanned (Bio-Rad) and analyzed by Delta 2D software (Decodon). The proteomic profile of proteins from nurse-like cells (NLC) was used as a reference map for spot analysis. Spots on 2D western blots were overlayed with the reference map, and matching spots were excised for protein identification by mass spectrometry. Preparation of peptide mixtures for MALDI-TOF-TOF and the MALDI-TOF measurement of spotted peptide solutions was carried out on a 4800 MALDI TOF/TOF™ Analyzer (Applied Biosystems) as described previously [41].

### Protein identification by LC–ESI–Ion trap analysis

For tryptic digestion, coomassie stained protein spot was cut into small pieces. Gel pieces were incubated and shaken with the following solutions: swelling solution (100 mM ammonium carbonate) for 5 min, shrinking solution (50 mM ammonium carbonate, 60% acetonitrile) for 30 min, swelling solution for 20 min and shrinking solution for 30 min. Gel pieces were dried by vacuum and rehydrated in 20 µl digestion buffer (10 ng/µl modified trypsin (Promega) in 50 mM ammonium carbonate, 10% acetonitrile). Samples were incubated at 37°C for 16 h. Tryptic peptides were extracted by incubating twice with 65% acetonitrile/5% formic acid for 30 min on the shaker and sonification for 5 min followed by incubation in 100% acetonitrile. All supernatants were collected in a new tube and dried under vacuum. Tryptic peptides were dissolved in 1.5 µl of 50% acetonitrile and diluted with 13.5 µl of 0.2% formic acid.

Identification was performed on an Agilent 1100 LC/MSD-trap XCT series system. The electrospray ionization system was the Chip Cube system using a Large capacity Chip (Agilent Technologies, Waldbronn, Germany). Sample loading (8 µL/sample) from the microtiter plate into the enrichment column was performed at a flow rate set to 4 µL/min with the mix of the two following mobile phases at a ratio 98∶2 (mobile phase A: 0.2% formic acid in H2O; mobile phase B: 100% ACN). LC gradient was delivered with a flow rate of 400 nL/min. Tryptic peptides were eluted from the reversed phase column into the mass spectrometer using a linear gradient elution of 2–40% B in 40 min. For MS experiments, following mode and tuning parameters were used: Scan range: 300–2000 m/z, polarity: positive, capillary voltage: 1900 V, flow and temperature of the drying gas were 4 L/min and 325°C. The MS/MS experiments were carried out in auto MS/MS mode using a 4 Da window for precursor ion selection, an absolute threshold of 10,000, after 3 MS/MS spectra, the precursor ion were excluded from fragmentation for at least one minute. The generic files for database searching were generated by Data Analysis software version 3.4, for precursor ion selection a threshold of 5 S/N was applied and the absolute number of compounds was restricted to 1000 per MS/MS experiment. Protein identification was performed with Mascot software[42]. MS/MS datasets were used to search the spectra against the human subset of the Swiss-Prot database[43].

### Immunoprecipitation of vimentin

To immunoprecipitate vimentin from protein extracts, 5 µg of the CLL BCR Ig014 were incubated for 3 hours at 4°C on a rocking platform with different protein extracts in a total volume of 500 µl (concentration of protein extracts 2 mg/ml). Per reaction, 20 µl of 50% sepharose-G slurry was washed in PBS twice, added to the reaction and incubated for another 30 minutes. Sepharose-G beads were pelleted at 500 rounds per minute (rpm) in a table centrifuge and washed twice with PBS. The beads were resuspended in sample buffer and subjected to one-dimensional SDS PAGE as described above. The presence of immunoprecipitated material, respectively vimentin, was determined by western blot analysis using Ig014 or an anti-vimentin antibody for detection.

### Immunofluorescence and confocal microscopy

M210B4 cells were seeded on coverslips (12 mm, Glaswarenfabrik Karl Hecht) in 24-well plates at a density of 1×10^5^ cells/ml. Cells were permitted to adhere and expand on the coverslip over one to three days until about 70% confluent. Cells were fixed with 4% paraformaldehyde and permeabilized with 0.5% Triton-X-100. The permeabilization step was omitted if antigens on the extracellular side of the plasma membrane should be detected. Fixed cells were blocked with 3% bovine serum albumin (BSA) in phosphate buffered saline (PBS). Stainings were performed using a rabbit anti-vimentin antibody (clone H-84, Santa Cruz Biotechnology) at 10 µg/ml and a secondary Fluorescein Isothiocyanate (FITC)-conjugated goat anti-rabbit IgG antibody (Santa Cruz Biotechnology) at 4 µg/ml. The CLL BCR Ig014 was used at a concentration of 100 µg/ml followed by a secondary FITC-conjugated rabbit anti-human IgG antibody (Sigma) diluted 1∶1000. Cell membranes and cytoplasm were counterstained with Alexa Fluor 594 phalloidin (Invitrogen) and mounted with VectaShield mounting medium (Vector Laboratories). Coverslips were fixed on a 76×26 mm slide (Roth). Images were obtained by confocal microscopy (Leika TCS SP2 AOBS; lens 63x) and analyzed using Leika confocal software.

To analyze the percentage of apoptotic cells, stromal cells were stained with 7-aminoactinomycin (7-AAD) on the immunofluorescence slides at different time points. Nuclei were counterstained with 4′,6-Diamidino-2-phenylindol (DAPI, Vector Laboratories). Images were obtained by conventional fluorescence microscopy (Carl Zeiss, Axioplan).

### Measurement of CLL apoptosis on feeder layers

CLL cells were cultivated alone or on a layer of M210B4 murine stromal cells blocked with either Ig014, the control BCR IgGr, or a commercially available rabbit anti-mouse/human vimentin antibody (clone H-84, Santa Cruz Biotechnology) at 100 µg/ml. The viability of CLL 014 cells was assessed after six days of co-culture by trypan blue staining or by fluorescence-activated cell sorting (FACS) using 7-aminoactinomycin (7-AAD) or (PI) and annexin V. For trypan blue staining, an aliquot of CLL cells was taken from the well, diluted 1∶10 in 0.4% trypan blue solution (Gibco) and trypan blue stained and unstained cells were counted after five minutes in a Neubauer counting chamber. Cell counts of four quadrants were used to calculate the percentage of viable cells. For FACS analysis, CLL cells were stained and measured according to the FITC Annexin V Apoptosis Detection Kit protocol (BD Pharmingen).

Alternatively, CLL cells were cultured on a feeder layer of vimentin secreting HEK 293T cells. Therefore, RNA was extracted from HEK 293T cells (using the Quiagen RNeasy kit) and cDNA was generated by reverse transcription using oligo(dT) primers (invitrogen). Subsequently, the vimentin encoding gene was amplified with gene-specific primers and cloned into the eukaryotic secretory expression vector pSectag2hygro C (invitrogen). HEK 293T cells were transfected with the vimentin encoding vector and vimentin secretion was verified by western blot analysis of the supernatant. CLL cell apoptosis was measured at different time points after co-culture on HEK 293T cells transfected with vimentin encoding pSectag2hygro C or the empty control vector.

## Results

### CLL B-cell receptors with common stereotypical configuration recognize stromal cell antigens

**Figure 1:**
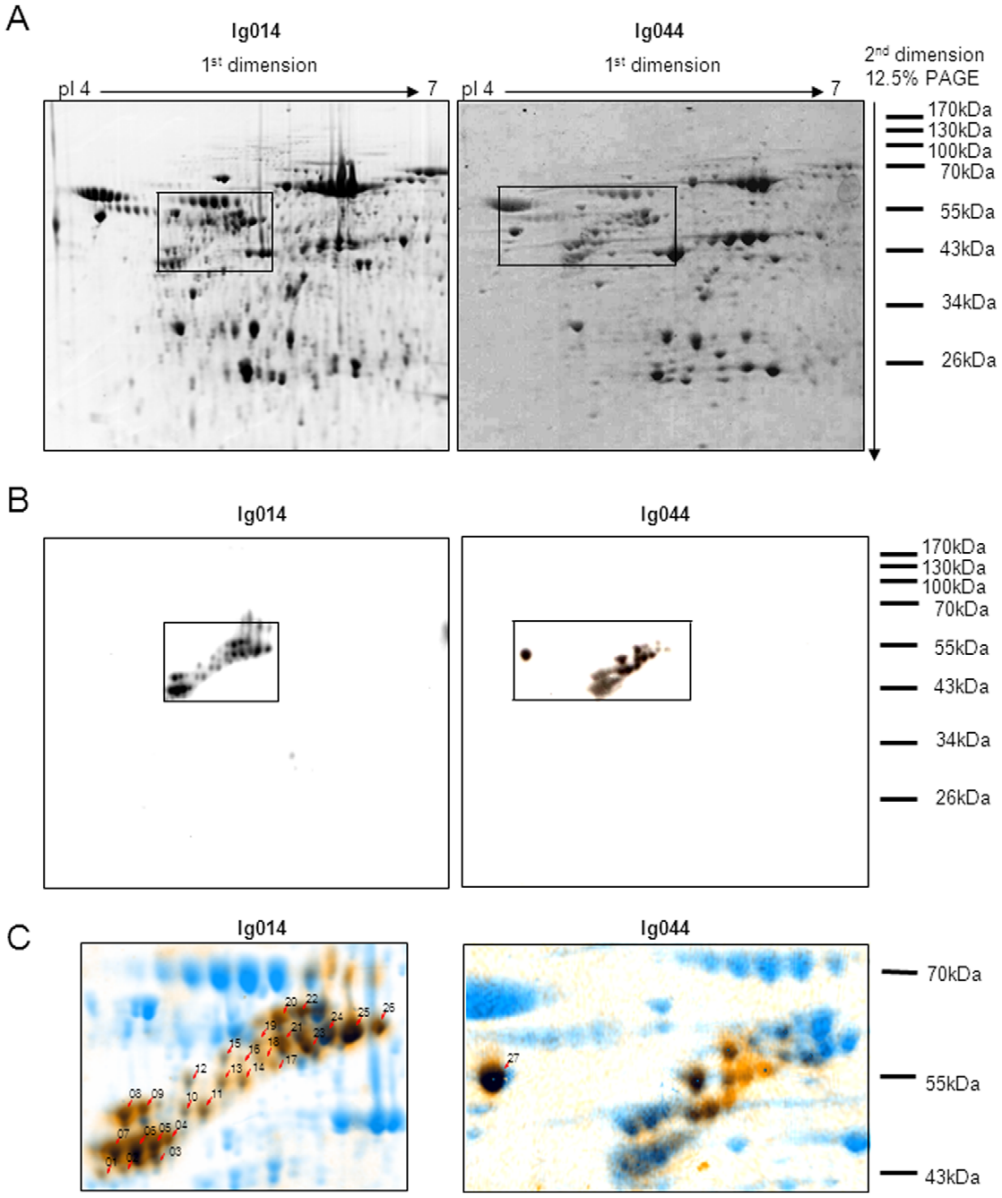
Subset 1 CLL B-cell receptors Ig014 and Ig044 recognize proteins highly expressed in nurse-like cell extracts. **A:** Two-dimensional gel electrophoresis of nurse-like cell protein extracts. Protein samples were prepared and electrophoresis was performed. The gel was subjected to Coomassie blue staining. pI  =  isoelectric pH value, M.Wt.  =  molecular weight. The rectangles indicate the areas magnified in C. **B:** CLL B-cell receptors Ig014 and Ig044 recognize partially overlapping protein spots in two-dimensionally separated nurse-like cell protein extracts. Gels run in parallel to the ones shown in Figure 1A were subjected to Western blotting using Ig014 and Ig044 as primary antibodies, followed by secondary detection with a horseradish peroxidase-conjugated antibody. A set of protein spots ranging from 45 to 57 kDa and a pI between 4.5 and 5.5 were detected by both Ig014 and Ig044. Another highly expressed protein spot (no. 27) was detected by Ig044, only. The rectangles indicate the areas magnified in C. **C:** Overlay of spots detected by Ig014 and Ig044, respectively, and the corresponding regions of the nurse-like cell protein map in Figure 1A. Delta 2D software (Decodon) was used for analysis. The magnified regions correspond to the highlighted regions from figure 1A and B. Overlapping spots are depicted in brown/black and marked by black arrows whereas spots which were only present on the Coomassie stained protein maps from Figure 1A are depicted in blue.

We tested the hypothesis that CLL B-cell receptors (BCR) may recognize antigens expressed on stromal cells. Therefore, we investigated the reactivity of a small set of recombinantly expressed CLL BCRs with protein extracts from stromal cells. All CLL BCRs used for this analysis expressed stereotyped heavy chain complementarity-determining region 3 (CDR3) sequences frequently found in CLL patients (subset 1, 3 and 7 according to Stamatopoulos [19] and Murray [38]). Table 1 summarizes BCR gene usage, CDR3 sequence, and mutational status of the four CLL BCRs used for this study. For protein extraction, we used primary nurse-like cells (NLCs) from the blood of CLL patients and the murine stromal cell line M210B4 alone or after co-culture with primary CLL cells. Extracted proteins of these cells were subjected to one-dimensional gel electrophoresis. The reactivity of the CLL BCRs Ig014, Ig015 and Ig022 as well as control IgG with proteins from the cell extracts was tested by Western blot analysis using the respective BCR as primary antibody, followed by HRP-based secondary detection. We found that the CLL BCR named "Ig014", which belonged to the stereotyped subset 1, recognized a range of proteins sized between 45 and 57 kDa that were expressed in the NLC extract and the stromal cell extracts (Figure S1). To identify the stromal proteins recognized by CLL BCR Ig014, the NLC protein extract was subjected to two-dimensional gel electrophoresis (Figure 1A). After electroblotting to a membrane and development with Ig014 and an HRP-conjugated secondary antibody, 26 spots were detected (Figure 1B, left Western blot) that corresponded in size to the bands detected in the one-dimensional gel electrophoresis. The isoelectric pH values (pI) of the detected spots ranged from 4.5 to 5.5. All protein spots were detectable in an independently performed two-dimensional electrophoresis by Coomassie blue staining, indicating that the proteins were abundantly expressed in NLCs. A magnified overlay of the Western blot and the corresponding Coomassie gel run in parallel is shown in Figure 1C (left panel) with all overlapping spots depicted in brown and black. The spots were excised and proteins were analyzed by mass spectrometry. Twenty-two of the 26 protein spots were identified as vimentin by MALDI TOF/TOF. The remaining four spots did not give any results, indicating that the amount of protein recovered from these spots was too low. The number and position of the detected spots reflect numerous posttranslational modifications of vimentin such as phosphorylation and citrullination, which have been previously described for this protein [44], [45], [46]. Table 2 shows protein scores and peptide counts for each analyzed spot.

**Table 1:** Characteristics of CLL B-cell receptors cloned as IgG1 antibodies.*

Ig Code	Isotype	VH Gene	VL Gene	HCDR3 Sequence	subset**	Mutation Status VH
Ig014	µ, κ	1-2	3-20	CARDQWLTLGNYFDYW	1	UM
Ig044	µ, κ	1-3	1-39	CARDQWLVLGMVFDYW	1	UM
Ig015	µ, λ	1-69	3-9	CARAVQEDYDFWSGYYPNYYYYGMDV	7	UM
Ig022	µ, κ	1-69	1-39	CAREQPDIVVVPADVGYYYGMDV	3	UM

*CLL denotes chronic lymphocytic leukemia, VH or VL are the genes used for the variable heavy or light chain of the BCR; HCDR3  =  heavy chain complementarity-determining region 3; M =  mutated; UM  =  unmutated.

**Subset of HCDR3 according to Stamatopoulos et al. (2007) and Murray et al. (2008).

**Table 2:** Identification of nurse-like cell proteins recognized by CLL BCRs Ig014 and Ig044 by mass spectrometry.*

S. No.	Detecting BCR	Protein Score	Protein Name	Protein MW	Protein PI	Peptide Count
2	Ig014, Ig044	**647**	Vimentin	53619	5.06	27
3	Ig014, Ig044	**553**	Vimentin	53619	5.06	27
4	Ig014, Ig044	**318**	Vimentin	53619	5.06	24
5	Ig014, Ig044	**641**	Vimentin	53619	5.06	28
6	Ig014, Ig044	**702**	Vimentin	53619	5.06	27
7	Ig014, Ig044	**753**	Vimentin	53619	5.06	28
8	Ig014, Ig044	**633**	Vimentin	53619	5.06	24
9	Ig014, Ig044	**594**	Vimentin	53619	5.06	29
10	Ig014, Ig044	**108**	Vimentin	53619	5.06	9
11	Ig014, Ig044	**618**	Vimentin	53619	5.06	28
12	Ig014, Ig044	**120**	Vimentin	53619	5.06	10
13	Ig014, Ig044	**634**	Vimentin	53619	5.06	32
14	Ig014, Ig044	**618**	Vimentin	53619	5.06	28
15	Ig014, Ig044	**123**	Vimentin	53619	5.06	8
17	Ig014, Ig044	**707**	Vimentin	53619	5.06	33
18	Ig014, Ig044	**582**	Vimentin	53619	5.06	27
19	Ig014, Ig044	**409**	Vimentin	53619	5.06	21
20	Ig014, Ig044	**815**	Vimentin	53619	5.06	31
21	Ig014, Ig044	**560**	Vimentin	53619	5.06	33
22	Ig014	**289**	Vimentin	53619	5.06	18
23	Ig014, Ig044	**565**	Vimentin	53619	5.06	23
24	Ig014	**661**	Vimentin	53619	5.06	27
27	Ig044	**371**	Calreticulin	48112	4.2	27

*S. No. denotes sample number, MW denotes molecular weight, PI denotes isoelectric pH.

To confirm that CLL BCRs using the stereotyped subset 1 HCDR3 region recognize vimentin, we tested the binding of another CLL BCR of this subset (Ig044) to the NLC protein extract. As indicated in Table 1, Ig014 and Ig044 express the same HCDR3 region whereas they differ in their heavy- and light-chain gene usage. Indeed, like Ig014, Ig044 recognized the spots previously characterized as vimentin (Figure 1A-C, right panels). In addition to these spots, Ig044 recognized another protein highly expressed in NLCs (Figure 1C, right panel, spot 27). This protein was identified as calreticulin by ESI-ion trap analysis (Table 2).

Because vimentin was shown to be recognized by both subset 1 antibodies, we focused further evaluation and functional studies on this antigen.

### Vimentin is highly expressed and can be detected in the supernatant of stromal cells

**Figure 2:**
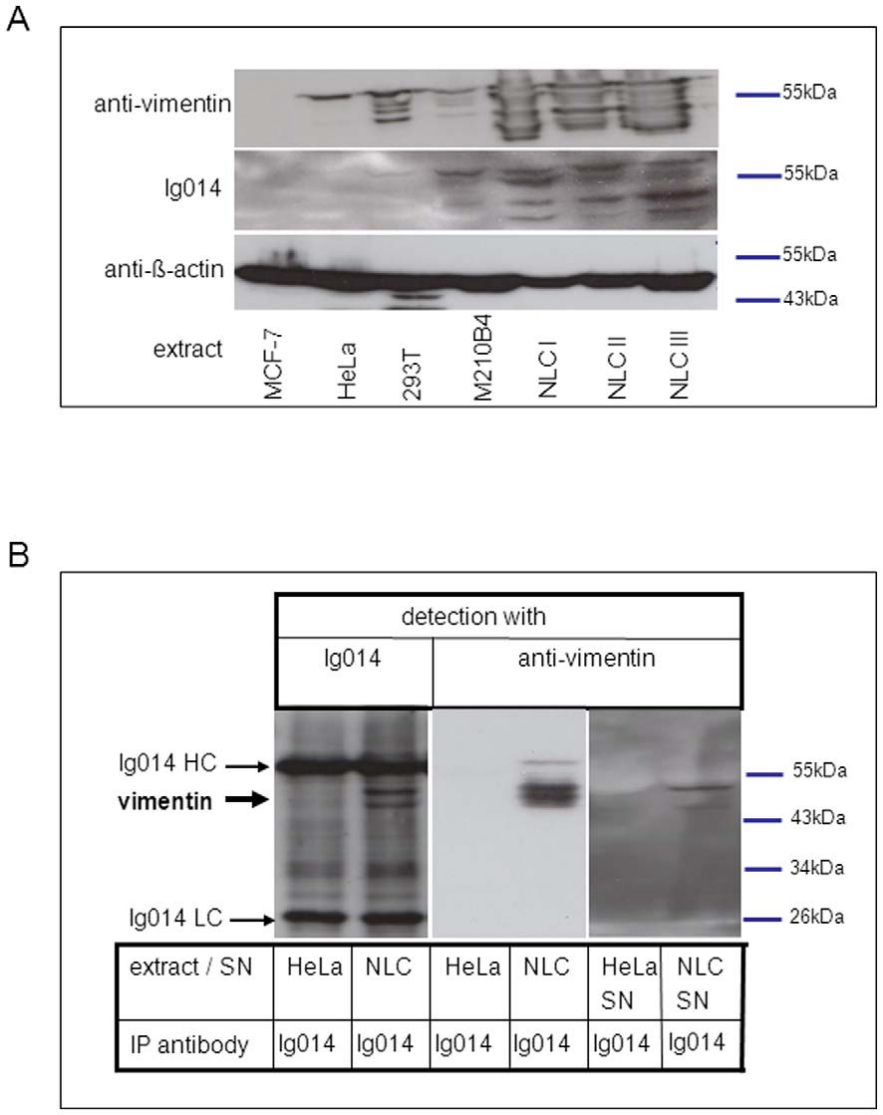
CLL B-cell receptor Ig014 recognizes and immunoprecipitates vimentin from stromal cell protein extracts and culture supernatants. **A:** Vimentin is recognized in various stromal cell protein extracts by recombinant CLL BCR Ig014 and a commercially available vimentin-antibody. Protein extracts were generated from human cell lines expressing no vimentin (MCF-7) or low levels of vimentin (HEK293T, HeLa) as well as protein extracts from the vimentin-expressing mouse stromal cell line M210B4 and from NLCs of three patients (I, II, III) with CLL. Protein extracts were separated by SDS-PAGE and subjected to Western blotting using Ig014 or a commercially available vimentin antibody for detection. A loading control was performed by staining with an anti-β-actin antibody. **B:** Vimentin can be immunoprecipitated from nurse-like cell protein extracts and supernatant. Immunoprecipitations were performed from a nurse-like cell extract (NLC) and supernatant (SN) using Ig014 and sepharose-G beads as described in the materials and methods section. A HeLa cell extract and HeLa supernatant was used as a negative control. Precipitates were separated by SDS-PAGE and subjected to Western blotting using Ig014 or a commercial anti-vimentin antibody as primary antibodies. Note that in the left panel the light (LC) and heavy chains (HC) of the Ig014 immunoglobulin used for immunoprecipitation is also detected because an anti-human immunoglobulin secondary antibody was used when Western blot staining was done with Ig014 as the primary antibody.

To corroborate the finding that vimentin functions as an antigen recognized by stereotyped CLL BCRs of subset 1, we further explored its interaction with a broader range of protein extracts from stromal and control cells and correlated it with the interaction pattern of a commercially available anti-vimentin antibody. We generated protein extracts from the following cell types: i.) human cell lines expressing no vimentin (MCF-7) or very low levels of vimentin (HEK293T, HeLa); ii.) the mouse stromal cell line M210B4 which is used to support primary human CLL cells in culture; iii.) primary NLC extracts from the peripheral blood of three patients with CLL. Protein extracts were separated by SDS-PAGE and subjected to Western blotting using Ig014 and a commercially available vimentin antibody as primary antibodies (Figure 2A). Detection with an anti-β-actin antibody revealed similar signals in each of the lanes, confirming that equal amounts of protein had been loaded. The soluble Ig014 BCR detected vimentin at the same pattern as a commercially available anti-vimentin antibody albeit at lower sensitivity. Vimentin was detected in all stromal cell-derived protein extracts, whereas a much weaker signal was obtained for HEK293T and HeLa cells, as expected (Figure 2A). Both antibodies did not detect specific signals in the protein extract from the vimentin-negative cell line MCF-7 (Figure 2A). The commercial anti-vimentin antibody showed a somewhat weaker signal for vimentin in the M210B4 mouse cell line extract which we attributed to the lower reactivity of this antibody with rodent vimentin than with human vimentin as described previously for this antibody [47]. Interestingly, Ig014 reacted much stronger with stroma-derived vimentin as compared to vimentin from other cell lines, possibly indicating that stroma-specific post-translational modifications could be preferentially recognized by this CLL BCR.

To prove that Ig014 interacts with vimentin also in its native conformation, immunoprecipitations of proteins from NLC protein extracts were performed. Ig014 was incubated with protein extracts from NLCs proven to express vimentin and then immunoprecipitated using protein-G sepharose beads. Precipitates were analyzed by Western blotting for the presence of vimentin using the Ig014 immunoglobulin or the commercial anti-vimentin antibody for detection. The blots showed clearly that Ig014 precipitated vimentin in its native conformation from NLCs. In contrast, no vimentin could be detected when control protein extracts were used for immunopreciptation (Figure 2B).

We reasoned that if stroma-derived vimentin serves as an antigen recognized by CLL cells, it is necessary that vimentin is at least partially expressed on the surface of and/or secreted and/or shed by stromal cells. In fact, it has been shown that macrophages can secrete vimentin and display it on their surface when activated by infectious agents [48]. To verify this for the CLL patient-derived NLCs, we immunoprecipitated both a supernatant from NLCs and a control supernatant from HeLa cells with Ig014. Vimentin was detected, albeit at low levels, in precipitates only if NLC supernatant was used for the immunoprecipitation (Figure 2B). No vimentin could be precipitated from the control supernatant.

### Vimentin is displayed on the surface of viable stromal cells

**Figure 3:**
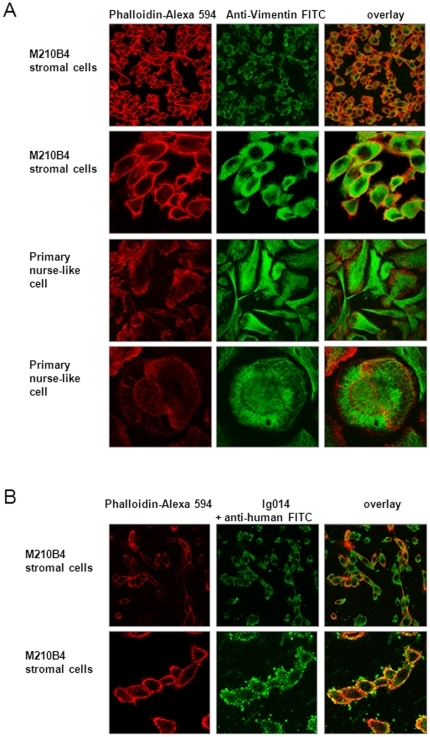
Stromal cells express high amounts of vimentin which can be immunostained with the CLL B-cell receptor Ig014. **A:** Visualization of vimentin expression in M210B4 stromal cells and nurse-like cells (NLC) by immunofluorescence confocal microscopy. M210B4 cells and NLCs were cultured on coverslips, fixed by paraformaldehyde and permeabilized by Triton-X-100. Vimentin was visualized using a FITC-conjugated anti-vimentin antibody (green). Cell membranes and cytoplasm were counterstained with Alexa Fluor 594 phalloidin (red). The right panel shows an overlay of both stainings. Stromal cells are shown at low (upper panel) and high magnification (intermediate panel). **B:** Visualization of Ig014 staining of M210B4 stromal cells by immunofluorescence confocal microscopy. Stainings were performed using Ig014 as primary antibody, followed by secondary detection with an anti-human FITC-conjugated antibody (anti-hu. FITC; green). Cell membranes and cytoplasm were counterstained with Alexa Fluor 594 phalloidin (red).

**Figure 4:**
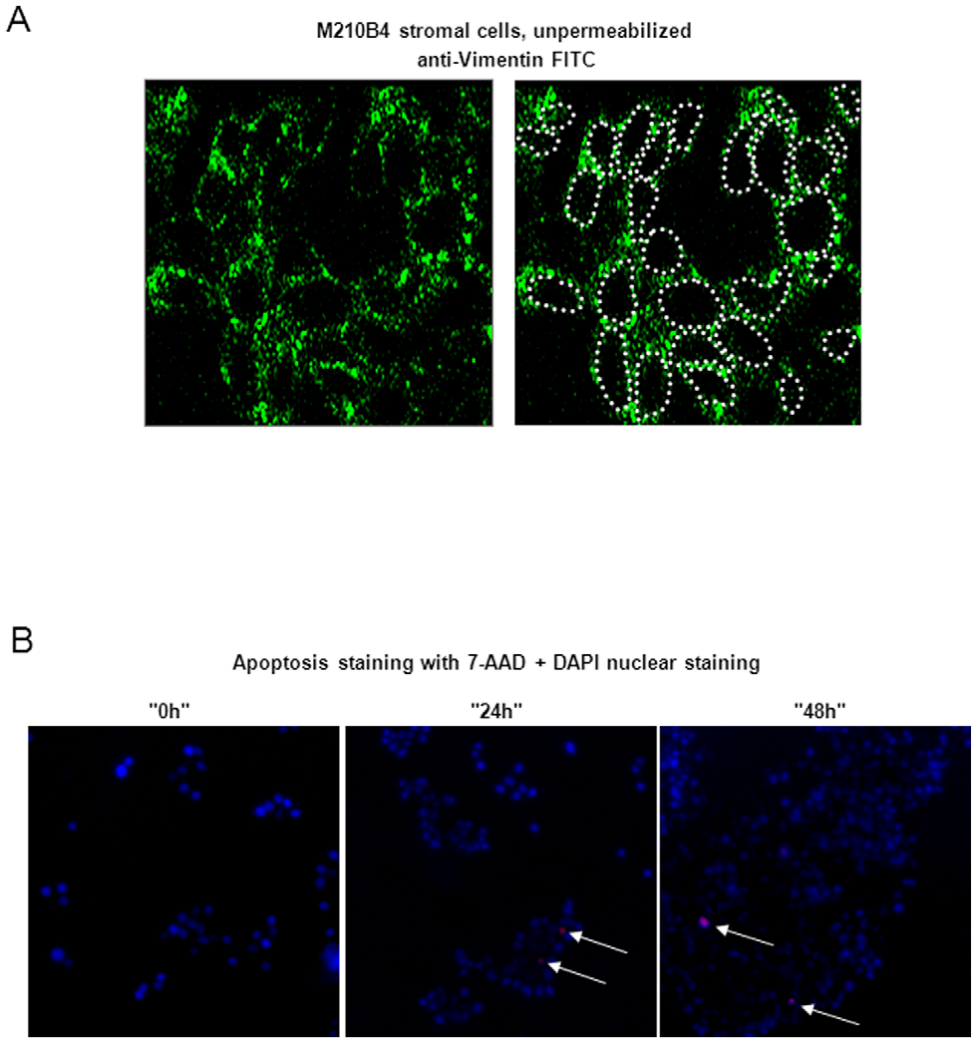
Viable stromal cells actively display vimentin on their cell surface. **A:** Vimentin is displayed on the surface of non-permeabilized M210B4 stromal cells as visualized by confocal microscopy. Cells were cultured and fixed with paraformaldehyde as above but omitting the permeabilization step. A FITC-conjugated Vimentin antibody stained the outer surface of the cells (green "corona") as shown in the left panel. Intracellular counterstaining could not be performed due to the non-permeabilized state of the cells. The right image highlights the cell margins by a dotted grey line. **B:** Stromal cells (as used for immunofluorescence stainings, Figure 4A) display vimentin independently of apoptotic events. Cell viability was assessed before the vimentin staining ("0 h"), as well as 24 and 48 hours later ("24 h" and "48 h"). Less than 1% of cells underwent apoptosis at time point "0 h" and "24 h" and less than 3% at time point "48 h" as demonstrated by staining with the apoptosis marker 7-AAD (interspersed purple cells, highlighted by white arrows). Nuclei were counterstained with DAPI (blue). Pictures were taken using conventional fluorescence microscopy.

The data described above shows that vimentin is present in the supernatant of NLCs which could be due to active secretion, membrane shedding, or as a result of NLC apoptosis. To investigate whether vimentin is indeed also detectable on the plasma membrane of stromal cells, we performed immunofluorescence stainings on NLCs and the mouse stromal cell line M210B4 using confocal microscopy. Both Ig014 and the vimentin antibody yielded a strong cytoplasmic and membrane signal in NLCs and M210B4 cells (green, Figure 3A and B) which partially matched the signal of phalloidin-Alexa 594 reacting with cytoplasmatic and membrane-bound actin (red, Figure 3A and B). To further determine whether the staining observed at the edges of the cells indicated the presence of vimentin on the extracellular side of the plasma membrane, we performed immunofluorescence on non-permeabilized stromal cells. Indeed, membrane-associated vimentin staining was also detected in non-permeabilized stromal cells (green "corona", Figure 4A), implying that some vimentin is displayed on the outer surface of the cell.

Regarding autoantigen-reactivity of CLL BCRs, it has been suggested that CLL emerges from B-lymphocytes that have been primed by (mainly intracellular) autoantigens released or displayed during apoptosis [33], [49]. This may also hold true for vimentin, that has been shown to be displayed on the surface of cells undergoing apoptosis [33]. We therefore investigated if the stromal cells staining positive for membrane-bound vimentin (almost 100% of all stromal cells) were viable at the time of staining or whether they were undergoing apoptosis at this point. Therefore, trypan blue and 7-aminoactinomycin (7-AAD) stainings were performed on the stromal cell layers used for immunofluorescence just prior to fixation. Of note, less than 1% of stromal cells stained positive for either trypan blue or 7-AAD, suggesting that the majority of cells were not apoptotic while displaying vimentin on the cell surface (Figure 4B). To exclude that the cells were in very early phases of apoptosis not yet detectable by trypan blue or 7-AAD staining, we also stained and counted the number of apoptotic/dead cells on the slide and in the supernatant 24 and 48 hours after the time point when the stromal cells were used for vimentin immunofluorescence stainings. We assumed that early apoptotic cells would then be in late phases of apoptosis and thus detectable in the supernatant and on the slide by stainings with apoptosis markers. After 24 hours, >99% and after 48 hours, >97% of the adherent stromal cells were viable and less than 100 dead cells (out of 10^5^ seeded adherent cells) were counted in the supernatant (Figure 4B, time points "24 h" and "48 h"). This indicates that at the time point of the vimentin staining, stromal cells were not undergoing apoptosis und thus vimentin was displayed “actively” on viable stromal cells.

### Stroma blocking with recombinant BCR reduces CLL 014 protection from apoptosis

**Figure 5:**
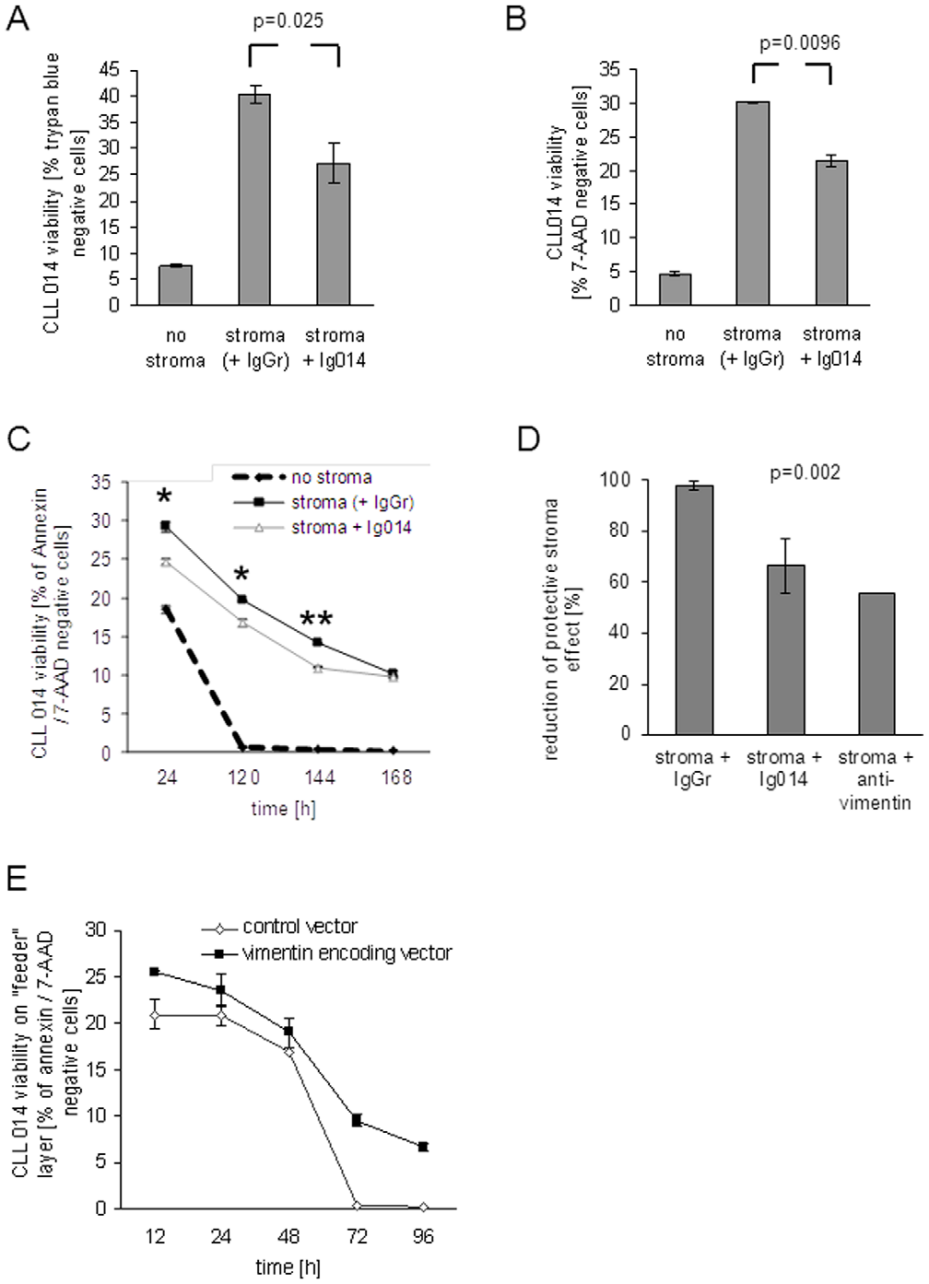
Stromal cell displayed or secreted antigen protects CLL 014 cells from spontaneous apoptosis. **A and B:** Soluble recombinant B-cell receptor Ig014 competes stromal cell-mediated CLL 014 cell protection from spontaneous apoptosis. CLL 014 cells were cultured alone or in co-culture with the stromal cell line M210B4. B-cell receptor-mediated stroma-CLL cell interactions were blocked by the recombinant B-cell receptor Ig014 or the control immunoglobulin IgGr. After six days of culture, CLL viability was assessed either by trypan blue staining (**A**) or by FACS analysis using 7-AAD (**B**). The diagrams show the percentage of viable CLL 014 cells at this time point (data are means of triplicates [A] or duplicates [B] +/− SEM). **C:** Time course of CLL 014 apoptosis on stroma blocked with the recombinant B-cell receptor Ig014 and with the control IgGr. Under the experimental conditions described in panel A and B of this figure, CLL 014 apoptosis was measured at different time points. To detect early and late phases of apoptosis, the measurement included staining with annexin V and 7-AAD followed by FACS analysis (Data are means of duplicates +/− SEM). **D:** Stromal cell-mediated CLL 014 cell protection can be competed by a known anti-vimentin antibody. CLL 014 cells were cultured alone, in co-culture with the stromal cell line M210B4 or with stroma cells blocked with either control B-cell receptor IgGr, CLL B-cell receptor Ig014, or a commercially available rabbit anti-mouse/human vimentin antibody. Apoptosis was measured after 12 hours of culture by staining with annexin V and PI followed by FACS analysis. CLL 014 cell viability reductions under blocked stroma conditions were calculated as relative values with unblocked stroma support (set to 100%). Data are means of duplicate experiments +/− SEM. **E:** A vimentin secreting "feeder layer" exerts a protective effect on CLL 014 cells. CLL 014 cells were cultured on a layer of HEK 293T cells transfected with a vimentin encoding secretory eukaryotic expression vector or an empty control vector. The viability of CLL 014 cells was determined at different time points of the co-culture experiment by FACS analysis using 7-AAD (data means of duplicates +/− SEM).

Stroma-mediated anti-apoptotic effects have so far been mainly ascribed to the action of cytokines and other anti-apoptotic proteins expressed by stromal cells. Having shown that the stroma-derived antigen vimentin is displayed on the surface of viable stromal cells, we asked whether this BCR-antigen interaction can protect CLL 014 cells from apoptosis. Therefore, CLL 014 cells were cultured alone and on a layer of M210B4 stromal cells blocked with either Ig014 or the control BCR IgGr at 100 µg/ml. The viability of CLL 014 cells was assessed after six days of co-culture by trypan blue staining and in a separate experiment by 7-AAD staining followed by fluorescence-activated cell sorting (FACS) analysis (Figure 5A and B). In both experimental approaches, the majority of previously cryopreserved CLL 014 cells lost viability after six days when cultured without a protective stromal cell layer (viability approximately 5%). There was a clear anti-apoptotic effect upon CLL cell cultivation on the stromal cell layer, resulting in 30–40% viable cells, when the stromal cell layer was blocked with the control BCR IgGr. When Ig014 was used to block the CLL-stroma interaction, the viability of CLL 014 cells was significantly lower (20–25% viable cells), suggesting an anti-apoptotic effect of the BCR-vimentin interaction on the surface or in the supernatant of M210B4 stromal cells. Next, CLL 014 cell viability was determined over a defined time course under the same conditions as specified above (Figure 5C). The measurement of apoptosis was performed by FACS analysis using 7-AAD complemented by annexin V to detect even earlier phases of apoptosis. Therefore, the percentage of viable CLL 014 cells was slightly lower than in previous experiments at a comparable time point. As in previous experiments, CLL 014 cells died rapidly without stromal support (Figure 5C). The anti-apoptotic effect of the stroma was significantly inhibited when stromal cells were blocked with the specific CLL BCR Ig014. This effect wore off after seven days of culture, when 90% of all co-cultured CLL cells were dead. To determine if the effect was indeed mediated by vimentin, we compared the inhibition of the protective stroma effect by BCR Ig014 with a commercially available anti-vimentin antibody. Therefore, CLL 014 cells were cultured alone, on a layer of M210B4 stromal cells, or on stroma cells and blocked with either control BCR IgGr, BCR Ig014, or the commercial anti-vimentin antibody with specificity for human and murine vimentin. The viability of CLL 014 cells was assessed after 12 hours of co-culture by Annexin/PI staining followed by fluorescence-activated cell sorting (FACS) analysis (Figure 5D). The protective stroma effect on CLL cells could be blocked to the same extent (approximately 40%) by both BCR Ig014 and the vimentin antibody as compared to unblocked or control antibody-blocked stroma (p = 0.002). These results suggest that vimentin is displayed on the surface of stromal cells and provides anti-apoptotic signals to CLL 014 cells through the BCR. We reasoned that, alternatively, the reduced viability of CLL cells on blocked stroma could be explained by the limited stroma-attachment itself, as a physical contact between stromal and CLL cells has been shown to be required for stroma-induced anti-apoptotic effects [7]. To dissect the effects of mere attachment-related and direct anti-apoptotic effects through the BCR, HEK 293T cells were transfected with a eukaryotic secretory expression vector encoding vimentin and an empty control vector, respectively, and used as a "feeder layer" for CLL 014 cells. HEK 293T cells transiently transfected with the vimentin encoding vector secreted vimentin in the supernatant (data not shown). When CLL 014 cells were cultured on this "feeder layer", the viability was enhanced compared to CLL 014 cells cultured on HEK 293T cells transfected with an insertless control vector (Figure 5E), suggesting protective effects of the secreted BCR antigen.

## Discussion

The B-cell receptor and its recognition of antigens may play a very important role in CLL pathogenesis and progression. Several antigens that are recognized by CLL B-cell receptors (BCRs) have been described. Yet, there is only speculation as to where these antigens are expressed, under which conditions they are exposed as well as whether – and if so: how – this relates to the interaction of the malignant lymphocytes with their microenvironment. Many studies have addressed the protective role of the stroma in CLL and different cell biological axes have been described to mediate these effects. These include protection from apoptosis by chemo- and cytokines, such as the stromal cell-derived factor-1 (SDF-1) which is secreted by stromal cells and interacts with the chemokine receptor CXCR4 on CLL cells [4]. After chemokine-mediated migration to the protective microenvironment, CLL cells attach to stromal cells via integrins, such as VLA-4 which binds to stromal VCAM-1 and fibronectin [14], [15]. Based on recent studies, however, there is reason to believe that stroma protection is not exclusively chemokine-, cytokine- and integrin-mediated but may also involve signaling through the BCR for antigen. Burger et al. recently showed that CCL3 and CCL4 expression is induced upon co-culture of CLL cells with stromal cells and that this can be blocked by antagonists of the BCR signaling pathway [34]. Moreover, there is evidence that antagonists of the BCR pathway, such as Syk or Akt inhibitors, can inhibit stroma-mediated protection from apoptosis [35], [50], [51], [52]. These data led us to hypothesize that stromal cells may constitute an antigen reservoir for CLL cells, thus providing not only cytokine- and classical adhesion molecule-mediated but also BCR-mediated survival signals.

To test this hypothesis, we cloned and expressed a set of CLL BCRs expressing common stereotyped HCDR3 sequences (subsets 1, 3 and 7 according to Stamatopoulos [19] and Murray [38]) and tested the interaction of these antibodies with lysates of a set of different cell types including stromal cells. We found that two CLL BCRs belonging to subset 1 [19], [38], termed Ig014 and Ig044, recognize the protein vimentin. One of the antibodies, Ig044, also interacted with calreticulin. The results of our confocal microscopy studies suggested that Ig014 interacts with vimentin exposed on the extracellular side of the plasma membrane of stromal cells. This BCR-mediated stromal antigen recognition seemed to be functionally relevant. We found a significant reduction of stroma-mediated protection from apoptosis in CLL 014 cells when stromal vimentin was competitively blocked by the CLL BCR or a commercially available anti-vimentin antibody and found an enhanced protective effect when feeder cells were forced to secrete vimentin. Therefore, our results prove for the first time that a stroma-derived antigen adds to the effect of stroma-mediated protection from apoptosis by BCR binding.

The CLL BCRs Ig014 and Ig044 use different heavy and light chain genes; yet, both of them are categorized as subset 1 antibodies [19], [38] as they express highly homologous HCDR3 sequences. Considering this, their common reactivity with vimentin is presumably mediated by their shared HCDR3, whereas calreticulin could be recognized by other regions of CLL BCR Ig044 such as CDR1, CDR2 or framework regions. Interestingly, vimentin and calreticulin have physiological and pathophysiological features in common. Both proteins are multifunctional, primarily intracellular proteins highly expressed in NLCs. Vimentin, a class III cytoskeletal protein in cells of mesenchymal origin, locates to the cytoplasm whereas calreticulin is expressed in the endoplasmic reticulum. Translocation to the plasma membrane has been described for both proteins [48], [53], [54], [55]. In addition to their physiological roles, vimentin and calreticulin are targets in autoimmune conditions, like rheumatoid arthritis [56], [57]. During apoptosis, vimentin can be exposed on the cell surface and triggers the generation of autoantibodies in some patients with rheumatoid arthritis [57]. Independently of apoptotic events, vimentin can be actively secreted and exposed on the extracellular side of the plasma membrane as it has been shown recently for activated macrophages [48]. Interestingly, macrophages and NLCs are similar cell types in that they both derive from monocytes. Vimentin has been described as an antigen recognized by different stereotyped (IGHV3-30-subset 32 [33], IGHV4-39-subset 8 [49]) and non-stereotyped (IGHV3-30 [33]) CLL BCRs. This finding has strengthened the hypothesis that CLL BCRs may frequently, if not generally, recognize molecular binding motifs on apoptotic cells, also taking into account reactivities with other proteins exposed on apoptotic cell surfaces. While this hypothesis may hold true, our data indicate that there may also be active secretion of proteins by viable stromal cells, potentially accounting for chronic antigen stimulation through the BCR.

CLL is a heterogeneous condition with some patients living for decades after diagnosis whereas others succumb rapidly to the disease despite therapy. Patients 014 and 044 of our study express a BCR with a stereotyped HCDR3 sequence ([19], [38]). This class of receptors is associated with an aggressive clinical course and effective treatment options are an unmet clinical need especially for this group of patients. As we improve our understanding of the protective role of accessory stromal cells in CLL, the microenvironment becomes an increasingly attractive biological target for novel treatment strategies in CLL.

## Supporting Information

Figure S1 **CLL B-cell receptor binding to protein extracts from stromal cells.** Primary nurse-like cells (NLCs) and the murine stromal cell line M210B4 alone or after co-culture with primary CLL cells were lysed and separated by one-dimensional gel electrophoresis. The reactivity of polyclonal IgG and the CLL BCRs Ig014, Ig015 and Ig022 was tested by Western blot analysis using the BCR as primary antibody followed by HRP-based secondary detection. Ig014 recognized a range of proteins between 45 and 57 kDa in size which were not recognized by the other BCRs.
(TIF)
