# Supplementary figures and images for: Stereotypical Chronic Lymphocytic Leukemia B-Cell Receptors Recognize Survival Promoting Antigens on Stromal Cells
Source: PLoS One. 2010 Dec 30;5(12):e15992. doi: 10.1371/journal.pone.0015992 (PMC3012720; doi:10.1371/journal.pone.0015992)

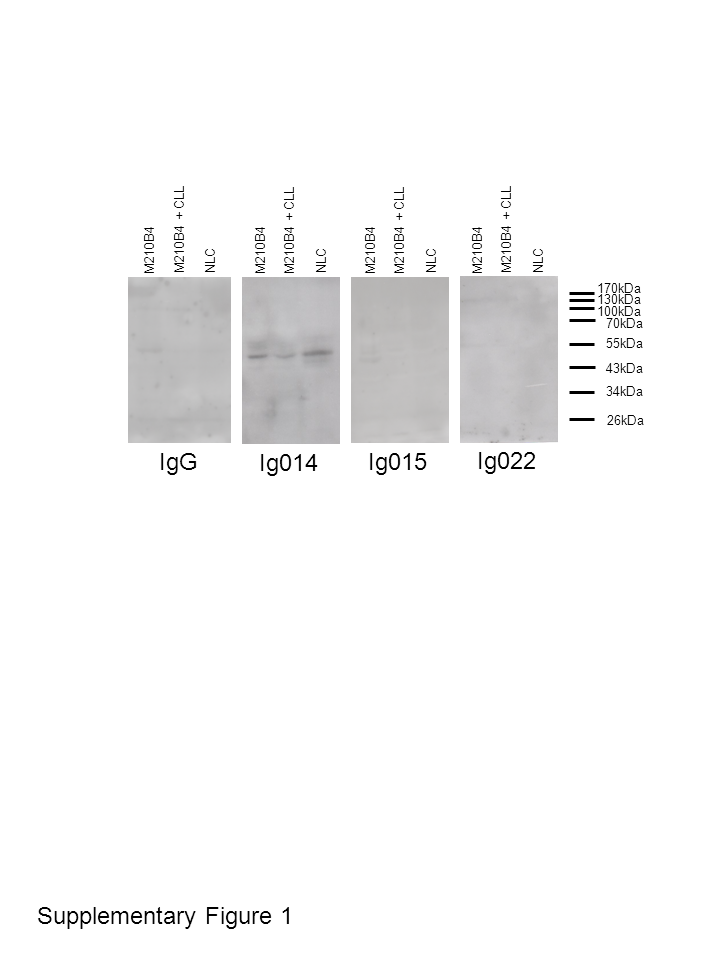

Supplement: Figure S1 — CLL B-cell receptor binding to protein extracts from stromal cells. Primary nurse-like cells (NLCs) and the murine stromal cell line M210B4 alone or after co-culture with primary CLL cells were lysed and separated by one-dimensional gel electrophoresis. The reactivity of polyclonal IgG and the CLL BCRs Ig014, Ig015 and Ig022 was tested by Western blot analysis using the BCR as primary antibody followed by HRP-based secondary detection. Ig014 recognized a range of proteins between 45 and 57 kDa in size which were not recognized by the other BCRs. (TIF) [file pone.0015992.s001.tif]
